# Oxymatrine Alleviates Collagen-Induced Arthritis in Mice by Regulating the Immune Balance of T Cells

**DOI:** 10.3390/molecules28155879

**Published:** 2023-08-04

**Authors:** Gan Cao, Jing Li, Zhuhan Mao, Yanli Zhang

**Affiliations:** Department of Pathogen Biology and Immunology, School of Basic Medical Sciences, Ningxia Medical University, Yinchuan 750004, China; caogan666@163.com (G.C.); ljjing0@163.com (J.L.); 20210210052@nxmu.edu.cn (Z.M.)

**Keywords:** gut microbiota, Th cell, oxymatrine

## Abstract

Rheumatoid arthritis (RA) is a chronic autoimmune disease characterized by systemic immunity and autoimmune disorders. We have previously found that oxymatrine (OMT), a natural alkaloid, can alleviate rheumatoid arthritis without knowing whether OMT can alleviate rheumatoid arthritis through gut microbiota. In this study, we found that OMT can alleviate collagen-induced arthritis in mice and reconstruct the immune balance of Th1/Th2, Treg/Th17, and Tfr/Tfh cells. Colon transcriptome gene enrichment analysis indicated that oxymatrine may alleviate collagen induced arthritis in mice through immune system process pathway. Furthermore, OMT significantly altered the gut microbiota variety, changed the composition of microbial colonies, and reshaped the gut microbiota of collagen-induced arthritis (CIA) mice, which may participate in the regulation of the balance of Th1/Th2, Treg/Th17, and Tfr/Tfh cells to alleviate collagen-induced arthritis in mice.

## 1. Introduction

Rheumatoid arthritis (RA) is a chronic systemic autoimmune disease characterized by progressive joint destruction, bone erosion, synovial hyperplasia, infiltration of immune cells, and the production of inflammatory factors [1]. Current pharmacologic treatments for RA include glucocorticoids, traditional nonsteroidal anti-inflammatory drugs, and new biological agents [2]. Most RA patients can achieve clinical remission after treatment with the above drugs, while about 30% of RA patients still suffer from adverse effects of the current treatment [3]. Methotrexate, as the most commonly used anti-RA drug in clinical practice, has hepatotoxicity when taken for a long time. Therefore, it is still necessary to find new treatments for RA.

At present, it has been found that various immune cells, such as dendritic cells, macrophages, T cells and B cells are involved in the pathogenesis of RA. Among them, the imbalance of CD4^+^T lymphocyte subsets is one of the key mechanisms of RA pathogenesis, and various T cell subsets and molecules related to T cell function are believed to be involved in the pathogenesis of autoimmune diseases. Previous studies have found that there is an imbalance in T cell subsets in the peripheral blood of patients with rheumatoid arthritis. But, the specific mechanism has not yet been fully elucidated.

Sophora flavescens has been reported to clear heat and remove dampness, which has antibacterial and anti-inflammatory effects [4]. OMT, as one of the major bioactive components in Sophora flavescens, has a wide range of pharmacological and biological activities [5], including antibacterial [6], antiviral, antioxidant, anti-tumor [7], and other effects [8]. Our team previously found that OMT can alleviate collagen induced arthritis by reducing the abundance of Tfh cells in the spleen, thereby reducing the production of autoantibodies. Therefore, OMT, with low toxicity and low side effects, is a potentially active compound that could be used in the treatment of RA.

Many studies have shown that the gut microbiota of RA patients is altered at the onset of the disease. In addition, the importance of the gut microbiota in the development of RA was also confirmed in collagen-induced arthritis mice. However, whether OMT can ameliorate RA by regulating gut microbiota and gene expression in the intestinal epithelium remains elusive. Regulating the homeostasis of gut microbiota to achieve the balance of CD4^+^T cell subsets may be a promising method for treating RA. Therefore, the purpose of this study is to explore whether OMT regulates the immune cells of RA through gut microbiota.

## 2. Results

### 2.1. OMT Alleviates Ankle Swelling, Arthritis Score, and Joint Histopathological Changes of CIA Mice

**Figure 1:**
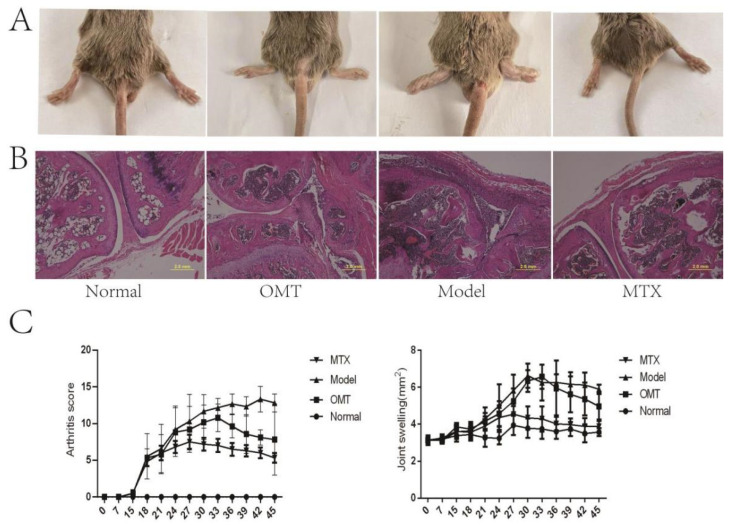
The severity of arthritis in CIA mice. (**A**) Representative image of mouse joint swelling. (**B**) H & E stained pictures of joints. (**C**) Changes in joint swelling and joint score of CIA mice.

Two weeks after secondary immunization, swelling of the paws and ankle joints was observed in collagen-induced arthritis mice, with some joints showing deformities in the CIA model group (Figure 1A). Compared with the control group, the degree of joint swelling (Figure 1C) and arthritis score (Figure 1C) of mice in the CIA model group were significantly increased, and the joint swelling of mice after OMT intervention was reduced. In the CIA model group, there was a significant proliferation of the synovial layer, significant articular cartilage damage, and infiltration of a large number of inflammatory cells in the articular cavity. The degree of inflammatory cell infiltration was significantly decreased in the OMT group and methotrexate group (Figure 1B).

### 2.2. OMT Treatment Reduces the Level of Inflammatory Factors

**Figure 2:**
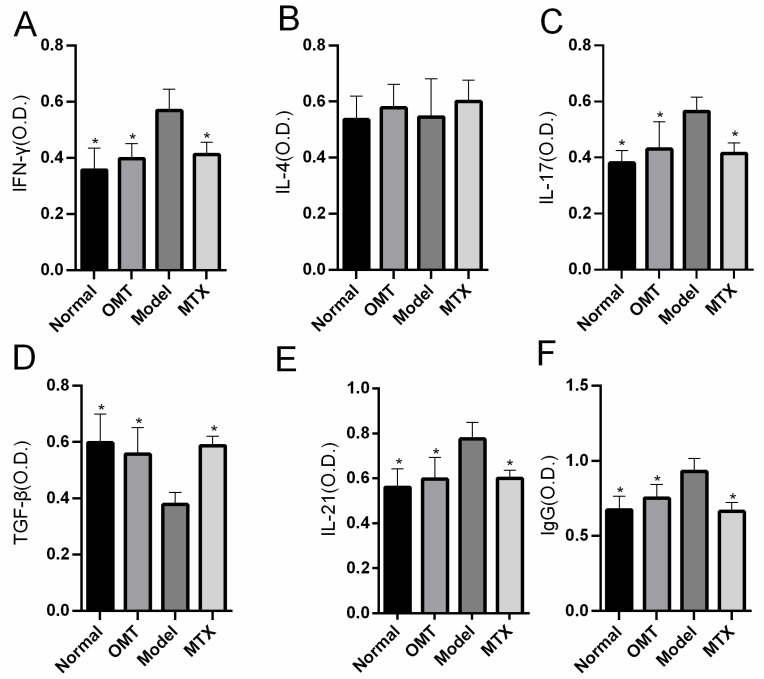
The effects of OMT on serum concentrations of (**A**) IFN-γ, (**B**) IL-4, (**C**) IL-17, (**D**) TGF-β, (**E**) IL-21, and (**F**) IgG. Data are expressed using mean ± SD. * *p* < 0.05 vs. the Model group.

The levels of IL-4, IFN-γ, IL-17, IL-21, TGF-β, and IgG in the serum of the mice were detected by ELISA. The results showed that the serum levels of IFN-γ, IL-17, IL-21, and IgG were significantly decreased (*p* < 0.05) and TGF-β was increased in the OMT group and the MTX group compared with the CIA model group, while there was no statistical difference in IL-4 between the OMT treatment group and the model group (*p* > 0.05) (Figure 2).

### 2.3. OMT Treatment Changes the Gut Microbiota Composition in CIA Mice

**Figure 3:**
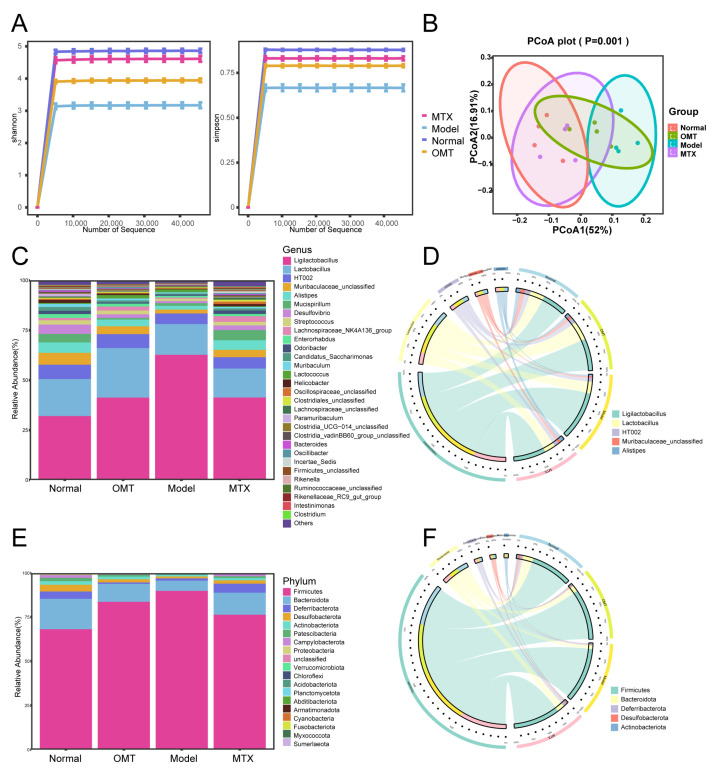
Composition analysis of intestinal flora. (**A**) Shannon and Simpson indices were used to estimate the differences in species diversity among the four groups. (**B**) The principal coordinate analysis (PCoA) results showed that the composition of the intestinal flora of the same group was similar, while the composition of the intestinal flora of different groups was different. (**C**) The relative abundance of intestinal bacteria at the genus level in the four groups. (**E**) The relative abundance of intestinal bacteria at the phylum level in the four groups. Circos diagram at the genus (**D**) and phylum (**F**) levels.

To study the regulation of OMT on the composition of gut microbiota in CIA mice, 16S rDNA gene sequencing was performed. According to the results of the Shannon and Simpson indices, the abundance of bacterial species in the CIA model group was lower than that in the normal group of mice, while the abundance of bacterial species in the OMT group and MTX group was higher than in the CIA model group (Figure 3A). The PCoA results showed a significant separation between the normal group, model group, MTX, and OMT groups (Figure 3B). Cluster analysis showed that the distance from the normal group to the OMT and MTX groups was shorter than the distance from the normal group to the model group. At the phylum level, *Firmicutes* and *Bacteroides* were the most abundant phylum among all samples (Figure 3E,F). In the model group, *Firmicutes* were higher than those in the control group, whereas *Bacteroidota, Patescibacteria,* and *Campylobacterota* were lower than those in the normal control group. OMT intervention reduced the abundance of *Firmicutes* and increased the abundance of *Bacteroidota, Patescibacteria,* and *Campylobacterota*. At the genus level, OMT decreased the abundance of *g_Ligilactobacillus* (Figure 3C,D).

### 2.4. OMT May Regulate Th Cells Imbalance through Immune System Processes

**Figure 4:**
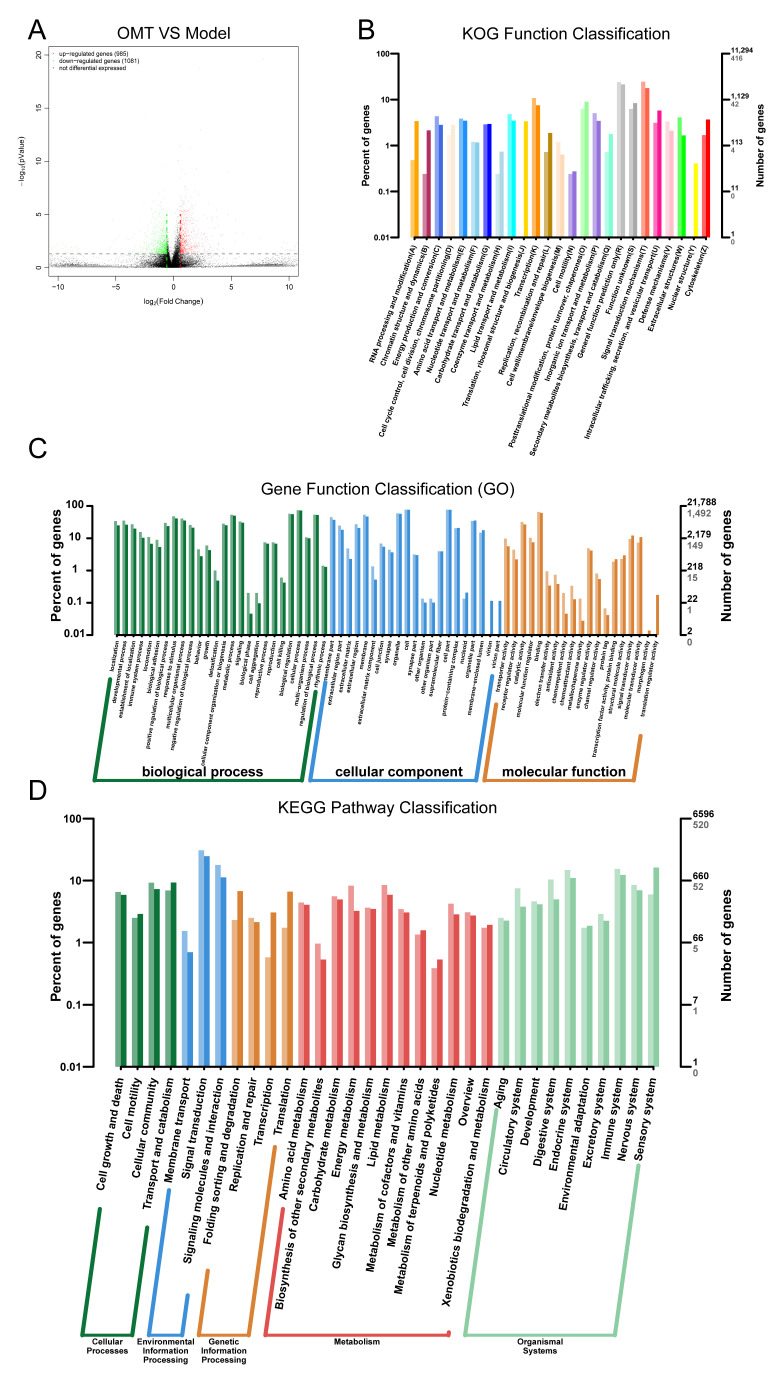
Transcriptomic analysis between the model and the OMT group. (**A**) Volcano map of differentially expressed genes (deg) between the two groups. (**B**) KOG enrichment analysis of differentially expressed genes. (**C**) GO enrichment analysis of differentially expressed genes. (**D**) KEGG enrichment analysis of differentially expressed genes.

We further studied the transcriptomics of the colon after OMT treatment to observe the mechanism of OMT in rebuilding the immune balance of CIA mice. As shown in Figure 4, a volcanic map (Figure 4A) was performed on the gene expression of the model group and OMT group. The KOG (Figure 4B), GO (Figure 4C), and KEGG (Figure 4D) enrichment analysis of differentially expressed genes was conducted, and most of the differentially expressed genes were enriched in the immune system processes pathway. Therefore, we speculated that OMT may regulate the balance of immune cells through the immune system processes.

### 2.5. OMT Could Balance Th Cells in CIA Mice

**Figure 5:**
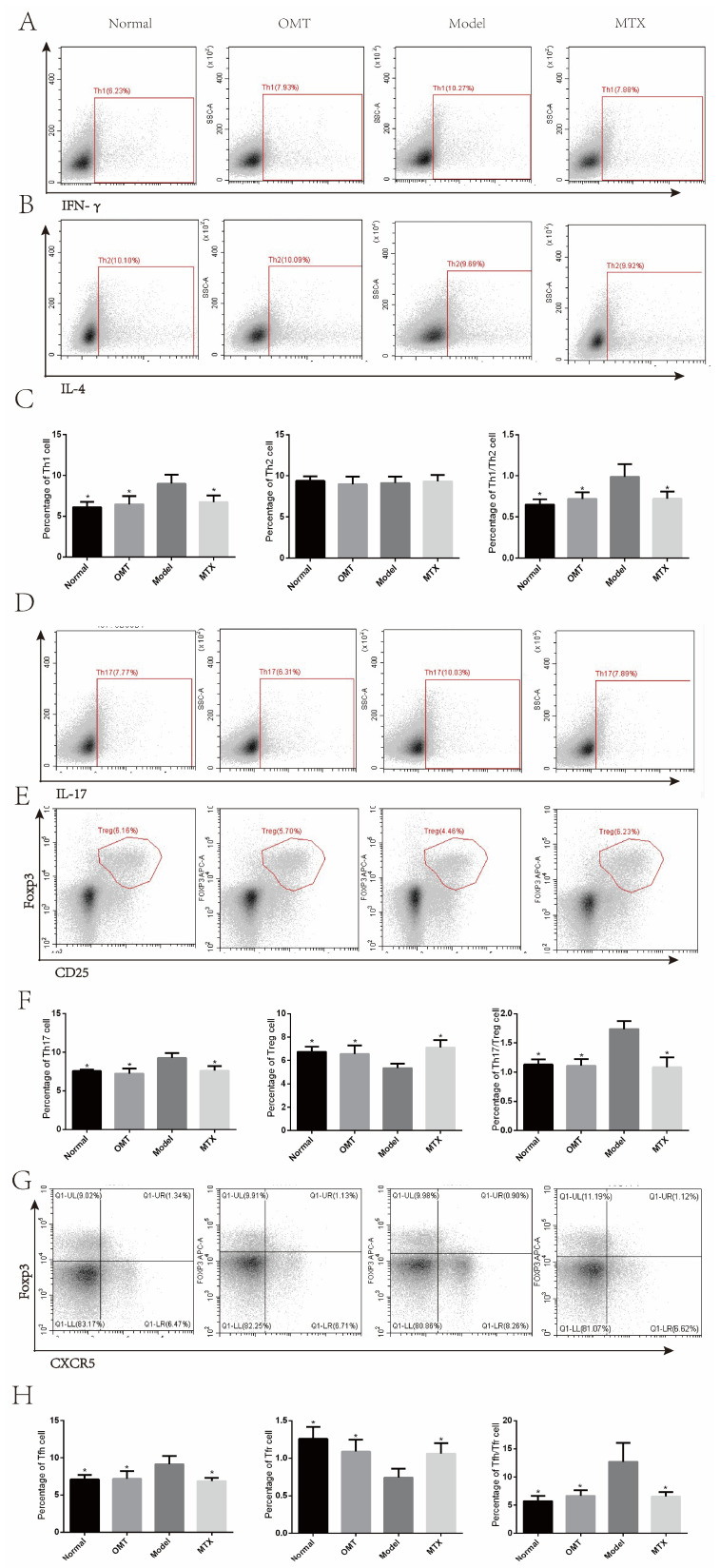
Flow cytometry was used to detect the expression of Th1/Th2, Treg/Th17, and Tfr/Tfh cells in mouse lymph nodes. (**A**–**C**) The percentage of Th1/Th2 cells in lymph node tissue was detected by flow cytometry. (**D**–**F**) Flow cytometry was used to detect the percentage of Treg and Th17 cells in lymph node tissue. (**G**,**H**) The percentage of Tfh/Tfr cells in lymph node tissue was detected by flow cytometry. Data are expressed using mean ± SD. * *p* < 0.05 vs. the model group.

In the CIA model group, the percentage of Th1, Tfh, and Th17 cells in the lymph nodes was significantly increased compared to the normal group, while the percentage of Treg, and Tfr cells decreased, and there was no difference in Th2 cells. In addition, there was an imbalance in Th1/Th2, Treg/Th17, and Tfr/Tfh cells (Figure 5). We assumed that the OMT group could reverse the imbalance of Th1/Th2, Treg/Th17, and Tfr/Tfh cells. The detection results of cytokines in serum are consistent with this result.

## 3. Methods

### 3.1. Animal and Reagents

DBA/1J mice were purchased from the Beijing Weitong Lihua Company (Laboratory Animal Production License No. SCXK (Jing) 2019-0008, Beijing, China). Mice were housed in a specific pathogen-free (SPF) environment at the laboratory animal center of Ningxia Medical University. OMT was purchased from Ningxia Bauhinia Flower Pharmaceutical Co., Ltd (Wuzhong, China). Bovine type II collagen, Freund’s complete adjuvant, and Freund’s incomplete adjuvant were purchased from Chondrex Company (Washington State, DC, USA). 

### 3.2. Establishment of Collagen-Induced Arthritis (CIA) Mouse Model

Bovine type II collagen was mixed 1:1 with Freund’s complete adjuvant, and 100 μL was injected subcutaneously into the tail of the mouse. After 21 days, bovine type II collagen was mixed 1:1 with Freund’s incomplete adjuvant, and 100 μL was injected subcutaneously into the back to induce the CIA mouse model.

### 3.3. Grouping and Administration

DBA/1J mice were randomly divided into four groups: Normal group, CIA model group, Oxymatrine (OMT) group, and Methotrexate (MTX) group with four mice in each group. From the 15th day after the first immunization, the normal group and the CIA model group were given 0.9% sodium chloride solution by gavage, and the dose of OMT group was 60 mg/kg/d, and it was continuously gavaged for 30 days. For the MTX group, the dose was 1 mg/kg/d, administered by gavage once every 3 days from the 15th day after the initial immunization. On the 45th day after the initial immunization, all mice were sacrificed by the cervical dislocation method, and peripheral blood, feces, lymph nodes, and colon were collected. Separation of lymph node lymphocytes was conducted using a Ficoll separation solution. The immune cells were analyzed by flow cytometry, and the feces were sent to Lianchuan Gene for 16S rRNA sequencing. The mouse colon was sent to Shanghai Shenggong for RNA-seq sequencing.

### 3.4. Evaluation of Rheumatoid Arthritis

From the day of initial immunization, the joint swelling and arthritis scores of the mice were measured and recorded once a week. The arthritis scores and joint swelling were measured every 3 days from the 15th day after the first immunization until the 45th day. Each measurement was performed by the same person using the same digital caliper to measure, score, and record the degree of swelling of the hind paws and ankle joints of the mice.

The evaluation criteria for the degree of inflammation are as follows: 0 = no redness or swelling, 1 = the small joint of the toe is red but not swollen, 2 = the toe joint is slightly red and swollen, 3 = the joint below the ankle joint is swollen, 4 = the ankle joint and feet are swollen with slight movement disorders, and the swelling degree of four feet is recorded and scored for each mouse. The maximum score for each mouse is 16.

### 3.5. HE Staining of Joint Tissues

On the 45th day after the first immunization, the mice were killed by cervical dislocation, and the joint tissue was taken out on the ultra-clean workbench, fixed in 4% Paraformaldehyde for 24 h, decalcified in formalin EDTA decalcification solution (G2520, Solarbio, Beijing, China) for one month, and replaced with fresh solution once a week. After complete decalcification, they were embedded in paraffin and sectioned at 4 μm. The sections were stained using a hematoxylin-eosin staining kit (Sl107-2, SEVEN, Beijing, China), and the infiltration of immune cells and vascular proliferation in joint tissue were observed under the microscope.

### 3.6. Analysis of CD4^+^T Cell Subsets in Mouse Lymph Nodes by Flow Cytometry

The lymph node was placed in a culture dish filled with PBS buffer (C3590-0500, VivaCell, Shanghai, China), and the lymph node was crushed into a single-cell suspension using a sterile rubber syringe head and filtered through a filter screen. The cell suspension was then added to a 15 mL centrifuge tube (601001, NEST Biotechnology, Wuxi, China) for centrifugation to obtain lymphocytes, and the cells were stimulated with 2 μL Cell Activation Cocktail (with Brefeldin A) for 4 h. Lymphocytes were resuspended in staining buffer (E-CK-A107, Elabsence, Wuhan, China) and labeled with Foxp3, IFN-γ, IL-4, or IL-17A, CD4, CD3, CD25, CXCR5, and the percentage of lymph node T cell subsets was analyzed on BECKMAN COULTER CytoFLEX.

### 3.7. Detection of Inflammatory Factors by Enzyme Linked Immunosorbent Assay (ELISA)

Store the serum in a −80 °C refrigerator for analysis. The levels of IL-4, IFN-γ, IL-17, IL-21, TGF-β, and IgG in the serum were detected using ELISA kits according to the manufacturer’s instructions (Shanghai Jianglai Industrial Limited by Share Ltd., Shanghai, China).

### 3.8. 16S rRNA Sequencing

Total DNA is extracted from mouse feces, followed by PCR amplification, recycling, purification, and examination by the Agilent 2100 Bioanalyzer (Agilent, Santa Clara, CA, USA). The qualified concentration should be above 2 nM. After gradient dilution of each qualified sequencing library, they were mixed according to the required sequencing amount in the corresponding proportion and denatured into a single chain through NaOH for sequencing. Using the NovaSeq 6000 sequencer, perform 2 × 250 bp double-ended sequencing with the corresponding reagent NovaSeq 6000 SP Reagent Kit (500 cycles).

### 3.9. RNA-Sequencing

The RNA was extracted from the mouse colon tissue, and the total RNA concentration and integrity were determined and then sequenced using the Huada Zhizao MGISEQ-2000. The quality of the sequenced raw data was evaluated through FastQC, and the original data were filtered. R language was used to analyze the gene expression difference. Subsequently, GO database enrichment analysis was conducted on differentially expressed genes (DEG), calculating the number of differentially expressed genes in each GO entry and then using hypergeometric testing algorithms to screen GO entries that were significantly enriched among differentially expressed genes. The KEGG statistical principle and method are similar to GO analysis.

### 3.10. Statistical Analysis

All data were expressed as mean ± standard deviation. Differences between two groups were analyzed by *t*-test, and differences between multiple groups were analyzed by one-way ANOVA. The data were processed using SPSS17.0 statistical software and GraphPad Prism8.0 software. *p* < 0.05 was considered statistically significant.

## 4. Discussion

Rheumatoid arthritis is an autoimmune disease characterized by swelling, hyperplasia of the synovium, and the production of a large number of autoantibodies, such as rheumatoid factor (RF) [9]. The interaction between microorganisms and the host immune system through microbial antigens and metabolites may disrupt the balance between the microbiome and the host immune system, leading to the onset of rheumatoid arthritis. Currently, an increasing number of clinical researchers have come to recognize the crucial role of gut microbiota in the progression and prognosis of rheumatoid arthritis. Studies have shown that its pathogenesis is mainly related to an imbalance in intestinal flora [10] and immune cells [11], as well as an imbalance in the immune regulation of related cytokines. In our study, we found that OMT alleviated the pathological manifestations of joint swelling, hyperplasia, and swelling of joint synovium and inhibited the expression of related inflammatory factors in CIA mice. The 16s rRNA sequencing results of feces showed that OMT reshaped the type and quantity of intestinal flora. In addition, colon transcriptome sequencing and flow cytometry results showed that immune system processes and CD4^+^ T cells played an important role in the mechanism of OMT relieving collagen-induced arthritis in mice.

In recent years, many researchers have explored the pathogenesis of RA from the perspective of gut microbiota, providing a new perspective and direction for clarifying the pathological mechanism of RA. Previous studies have shown that the gut microbiota homeostasis imbalance stimulates the mucosal immune disorder and destroys the self immune tolerance, leading to the imbalance of T cell subsets in the body, thus releasing inflammatory cytokines and promoting the occurrence of RA. In rheumatoid arthritis patients, Chen’s findings [12] found that multi-omics profiling reveals potential alterations in rheumatoid arthritis with different disease activity levels. In terms of animal experimental research, Qin et al. [13] found that Toddalia asiatica extract attenuates adjuvant-induced arthritis by modulating colon Th17/Treg balance and colony homeostasis. Lai et al. [14] found that Lycium barbarum polysaccharide modulates gut microbiota to alleviate rheumatoid arthritis in a rat model.

Gut microbiota may act as regulatory mediators and are factors that affect the immune system [15]. There is currently a debate about whether the diversity of gut microbiota in patients with rheumatoid arthritis has changed. Some studies have shown that the changes in the diversity of gut microbiota [16] in different stages of disease progression in rheumatoid arthritis are different. In this study, the feces of mice were analyzed by 16S rDNA sequencing, and it was found that the diversity of gut microbiota in the CIA model group mice was lower than that in the normal group of mice, while β diversity analysis showed significant differences in PCoA among different groups.

Analysis of species abundance at the phylum and genus levels of intestinal microflora in each group showed considerable variability. At the phylum level, *Firmicutes*, *Bacteroidota*, *Deferribacterota*, and *Desulfobacterota* are the main phylum of the intestinal microbiota in each group. Our results showed that OMT treatment ameliorated the imbalance of intestinal microflora, including bacteria such as *Firmicutes* and *Bacteroidota.* The cell wall of *Firmicutes* is composed of a relatively thick (10–50 nm) layer of peptidoglycans containing cell wall acids, most of which are gram-positive bacteria, and *Bacteroides* is a gram-negative bacterium. As a normal flora of the intestine, studies have shown that *Firmicutes* in the intestine can more effectively absorb heat from food, leading to obesity. In our study, we found that the abundance of *Firmicutes* increased in CIA mice while the abundance of *Bacteroides* decreased, indicating that the intestinal flora of CIA mice was dysregulated. This is consistent with the results of the trial of trioxide [17]. Interestingly, the ratio of *Firmicutes* to *Bacteroides* could be adjusted by OMT treatment. These results suggested that the imbalance of intestinal flora in CIA mice could be regulated through OMT.

CD4^+^ T cells play an important role in the pathogenesis of RA [18]. In our study, changes in fecal microbiota may mediate immune system processes to regulate the proportion of CD4^+^T cell subsets. Th1, Th2, Th17, and Treg cells are different subsets of T lymphocytes that participate in the pathological process of RA [19]. Therefore, it is generally believed that the imbalance between Th1/Th2, Th17, and Treg can lead to the onset of RA. Our results showed that there was also an imbalance between Tfh cells and Tfr cells and their related inflammatory factors IL-21, TGF-β. The imbalance of CD4^+^T cell subsets in CIA mice plays a critical role in the pathogenesis of rheumatoid arthritis [20]. In order to confirm whether the CD4^+^T cell subsets in CIA mice are unbalanced, we measured the levels of Th1/Th2, Th17/Treg, Tfr/Tfh, and their related cytokines in each group of mice. The results showed that, compared with the normal group, the number of Th1, Th17, and Tfh cells in the lymph nodes of the CIA model group significantly increased, while the number of Treg and Tfr cells significantly decreased.

Th1 cells and their secreted IFN-γ [21], IL-2, and other proinflammatory cytokines mediate cellular immunity, while Th2 cells mainly mediate humoral immunity [22]. Previous studies have shown that there is a significant imbalance in CD4^+^ T cell subsets in RA patients and collagen-induced arthritis models, with Th1 cell subsets being significantly dominant and corresponding cytokines significantly increasing, leading to chronic inflammation in RA. The relationship between Th17 and Treg cells is complex and functionally antagonistic to each other [23]. Th17 cells promote inflammatory reactions, while Treg cells have anti-inflammatory effects and maintain immune tolerance. Therefore, the imbalance between Th17 and Treg cells is closely related to the development of RA [24,25].

Follicular helper T cells and follicular regulatory T cells play an important role in the response to GC and in regulation of B cells [26]. Tfh cells promote B cell activation, proliferation, and differentiation, as well as plasma cell formation [27]. Tfr cells inhibit the function of Tfh cells and B cells and prevent the production of autoreactive antibodies [28]. In RA patients, abnormal GC B cell responses can often be observed, manifested by the presence of a large number of autoreactive antibodies in body fluids and synovium, as well as increased secretion of Tfh cells and IL-21. This indicates that the regulatory function of Tfr cells is abnormal, indicating that the imbalance in the interaction between Tfh cells and Tfr cells may play an important role in the occurrence and development of RA.

We acknowledge that although we have demonstrated that OMT regulates the imbalance of Th cell subsets through gut microbiota, the specific molecular mechanism of regulation has not yet been clarified. We need to conduct more in-depth research in the future to explore the specific gut microbiota involved in the process of oxymatrine relieving rheumatoid arthritis and its potential role in regulating the immune response.

In summary, the proportion of gut microbiota and CD4^+^T cell subsets in CIA mice was seriously unbalanced, and the flora diversity was reduced in the transition from a stable state to an “inflammatory” state. After OMT was administered, the flora abundance and CD4^+^ T cell subsets of CIA mice were closer to the level of normal mice, and joints were effectively restored. The results of gene enrichment analysis of colon transcriptome showed that the immune imbalance of T cells may be related to the immune system process, but the specific signal pathway has not been fully clarified, which needs further discussion.

## 5. Conclusions

Our data suggest that OMT may regulate the imbalance of Th cell subsets through intestinal microflora, thereby inhibiting GCB cells, reducing the production of autoantibodies, and alleviating the immunopathological process in CIA mice. It enriches the clinical treatment strategies for RA.

## Data Availability

The data used to support the results of this study are included in this article.

## Sample Availability

Samples of the compounds are not available from the authors.
